# Modeling the Impacts of Weather and Cultural Factors on Rotundone Concentration in Cool-Climate Noiret Wine Grapes

**DOI:** 10.3389/fpls.2019.01255

**Published:** 2019-10-15

**Authors:** Andrew D. Harner, Justine E. Vanden Heuvel, Richard P. Marini, Ryan J. Elias, Michela Centinari

**Affiliations:** ^1^Department of Plant Science, The Pennsylvania State University, University Park, PA, United States; ^2^Horticulture Section, School of Integrative Plant Science, Cornell University, Ithaca, NY, United States; ^3^Department of Food Science, The Pennsylvania State University, University Park, PA, United States

**Keywords:** climate, terpenes, pepper aroma, predictive model, regression analysis, *Vitis* hybrid

## Abstract

The sesquiterpenoid rotundone is the compound responsible for the “black pepper” aroma of many plant species, including several economically important wine grape varieties. Since its identification in wine in 2008, there has been an increased interest in understanding how individual climatic or cultural factors affect the accumulation of rotundone in grapes and subsequently the level of wine “pepperiness.” However, no study has assessed climatic and viticultural factors together to identify which variables have the strongest influence on rotundone accumulation. Our study aimed to fill this knowledge gap by developing a predictive model that identified factors that explain rotundone concentrations in Noiret (*Vitis* sp.) grapes at harvest. Over the 2016 and 2017 seasons, we measured 21 viticultural, meso- and microclimatic variables and concentrations of rotundone in Noiret wine grapes at seven vineyards in the northeastern U.S. Vineyard growing degree days (GDD*_v_*) and the amount of solar radiation (cumulative solar exposure; CSE_v_) accumulated from the beginning of fruit ripening to harvest were the variables best correlated (*r* = 0.70 and *r* = 0.74, respectively) with rotundone concentrations. Linear correlations between microclimatic parameters and rotundone concentrations were weaker, but overall rotundone was negatively correlated with low (<15°C) and high (>30°C) berry temperatures. Using the 2-year data set we were able to develop a four-variable model which explained more than 80% of the variation in rotundone concentration at harvest. The model included weather [growing degree days during fruit ripening (GDD*_v_*)] and plant-related variables (concentrations of phosphorus and calcium in the leaf petiole, and crop load). The model we developed could be used by wine producers to identify sites or cultural practices that favor rotundone accumulation in Noiret grapes after performing a model validation with an additional, external data set. More broadly, the statistical approach used here could be applied to other studies that also seek to assess the effects of multiple factors on a variable of interest under varying environmental conditions.

## Introduction

Aroma impact compounds and their interactions are an essential component of wine quality as they can contribute to pleasant or unpleasant wine sensory attributes. The most recent aroma impact compound identified in grapes is the sesquiterpene rotundone (C_12_H_22_O), which is responsible for the key “black pepper” aroma of Shiraz (*Vitis vinifera*) wines (Wood et al., 2008). Rotundone is a strong aroma compound, with a sensory detection threshold of 16 ng/L in red wine (Wood et al., 2008). It accumulates mainly within the berry exocarp beginning at the onset of fruit ripening (i.e., veraison) until harvest (Zhang et al., 2016). Since its first extraction from Shiraz grapes and wine, it has been identified in other red-fruited *Vitis vinifera* varieties across many wine-producing regions (e.g., Duras, Gamay, Mourvèdre, Durif, and Vespolina; Wood et al., 2008; Caputi et al., 2011; Geffroy et al., 2014) and to a lesser extent in white-fruited *V. vinifera* varieties (Grüner Veltliner; Caputi et al., 2011). Most recently, rotundone was extracted from grapes and wine of a red-fruited *Vitis* interspecific hybrid variety, Noiret (Homich et al., 2017).

It is still unclear why some grapevine varieties produce rotundone either at low or high concentration while others do not; like other sesquiterpenes, rotundone could be involved in the chemical communication between plants and other beneficial or pest organisms (Dunlevy et al., 2009). To date, studies mainly focused on identifying environmental factors responsible for rotundone accumulation in the fruit which will ultimately influence the “peppery” intensity of the wine (Geffroy et al., 2014; Zhang et al., 2015a; Bramley et al., 2017). Similar to other aroma impact compounds (e.g., 3-isobutyl-2-methoxypyrazine, or IBMP), rotundone accumulation in grapes depends upon climatic factors. Rotundone concentration in *Vitis vinifera* and hybrid varieties was positively associated with cooler temperatures (i.e., cool vintages or cool sites; Herderich et al., 2015; Homich et al., 2017). Grapes grown in shade, whether due to vineyard row orientation, cluster position within the vine canopy, or berry position within an individual cluster, had higher concentrations of rotundone when compared to grapes grown with higher solar exposure (Herderich et al., 2015). The decreased rotundone concentrations were attributed to the negative, direct effects of solar radiation, to the increased temperatures of berries with high sun exposure, or their combination.

Less clear is the influence of cultural practices on rotundone accumulation. Fruiting zone leaf removal, a popular canopy management strategy, has been the most studied because it influences fruit sun exposure and temperature, but contrasting effects on rotundone accumulation were reported depending on timing and severity of its application (Geffroy et al., 2014; Homich et al., 2017). Environmental and viticultural factors might operate in tandem to determine rotundone concentration in the fruit and “peppery” intensity of the wine. Understanding the relative importance of these variables on rotundone concentration may help clarify which sites or viticultural management methods are more conducive to producing wines with a desired level of pepperiness.

This study addresses this knowledge gap and incorporates a multitude of environmental, viticultural, and physiological data to assess which variables have the greatest influence on rotundone concentrations within Noiret wine grapes. The objectives of this 2-year study were to identify the key climatic and viticultural variables that influence rotundone concentration in Noiret grapes using seven vineyards with varying weather conditions; and to investigate the relationships between fruit sunlight exposure, berry temperature, and rotundone accumulation in Noiret grapes at harvest. More broadly, this work provides insights into how multiple regression statistical methods can be used within a horticultural context to assess relationships between factors associated with both plants and the environment to identify which factors may be of the utmost importance for the topic at hand.

## Materials and Methods

### Description of Experimental Sites

**Figure 1:**
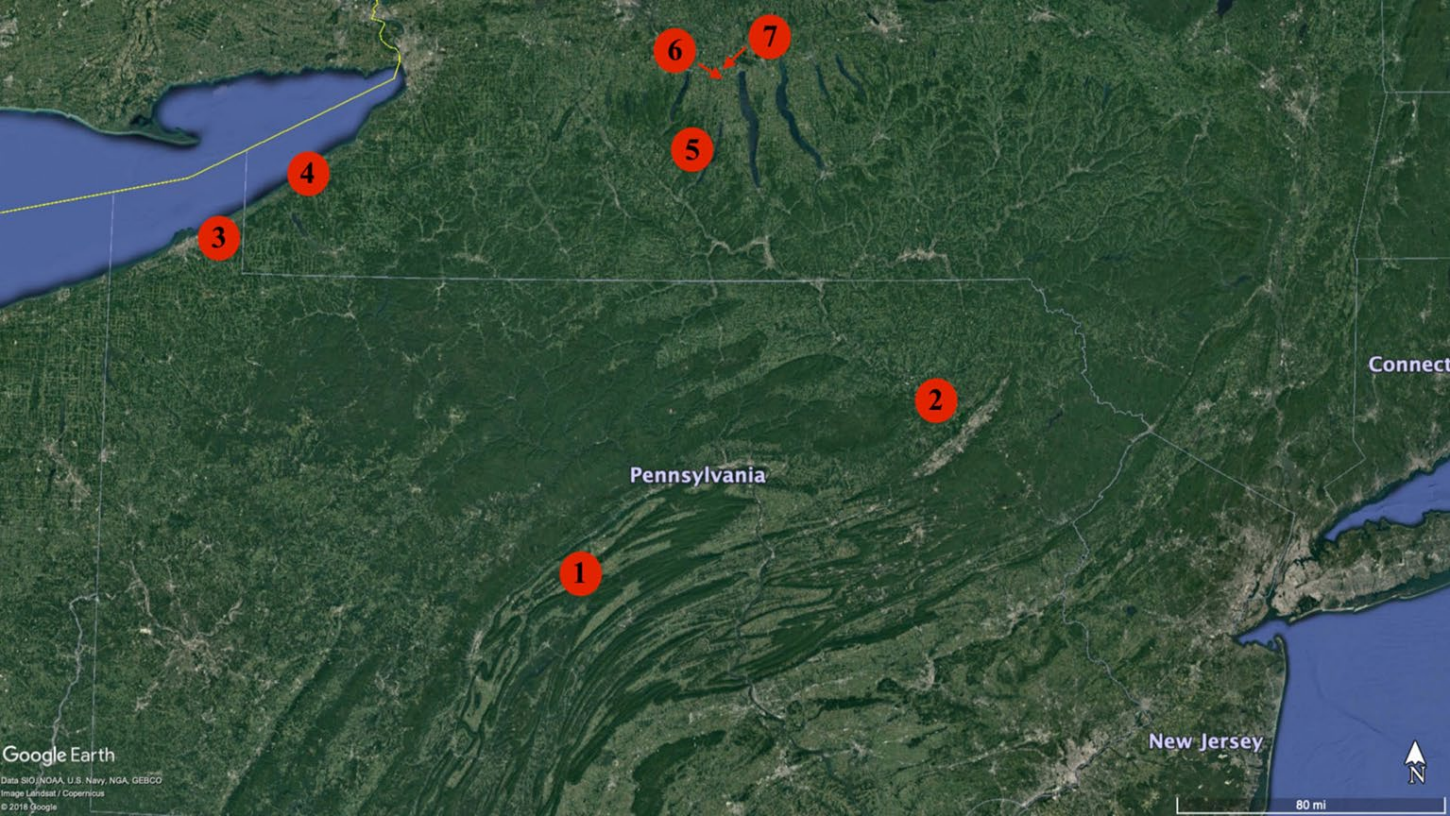
Map of Noiret vineyards selected for the study. A circle was imposed at the geographical coordinates of each study site. Geographical coordinates of each site are: Site 1: 40º47′N; 77º51′W; Site 2: 41º46′N; 75º84′W; Site 3: 42º22′N; 79º78′W; Site 4: 42º37′N; 79º47′W; Site 5: 42º58′N; 77º17′W; Site 6: 42º86′N; 77º03′W; Site 7: 42º88′N; 77º01′W.

The study was conducted in 2016 and 2017 at seven Noiret (*Vitis* hybrid cross of NY65.0467.08 and Steuben) vineyards located in the U.S. states of Pennsylvania and New York (**Figure 1**). The three Pennsylvania vineyards included three commercial vineyards located in State College (Site 1), Falls (Site 2), and North East (Site 3; **Table 1**). In New York there were two commercial vineyards in Portland (Site 4) and Branchport (Site 5), and two research vineyards at the Cornell University AgriTech in Geneva (Site 6; Site 7). Sites 5, 6 and 7 were in the Finger Lakes American Viticultural area (AVA). Information regarding vineyard age, vine and row spacing, training system, rootstock, and soil series classification is summarized in **Table 1**. Disease, pest, and canopy management practices (e.g., shoot training, thinning, and trimming) were performed by the grower cooperator in accordance with standard commercial practices for hybrid *Vitis* varieties in the eastern U.S. (Wolf, 2008).

**Table 1:** Location and vineyard information for the Noiret sites used in the study.

Site	Treatment^a^	Location	Rootstock	Spacing (m/row x m/vine)	Training^b^ system	Vineyard age^c^	Soil series^d^
1	C	State College, PA	101-14 Mgt	1.83 x 2.44	HWC	10	Hublersburg silt loam
1	LR	State College, PA	101-14 Mgt	1.83 x 2.44	HWC	10	Hublersburg silt loam
2	C	Falls, PA	Own-rooted	1.83 x 2.44	VSP	15	Lordstown channery silt loam
2	LR	Falls, PA	Own-rooted	1.83 x 2.44	VSP	15	Lordstown channery silt loam
3	C	North East, PA	Own-rooted	1.83 x 2.44	VSP	7	Chenango gravelly silt loam
3	LR	North East, PA	Own-rooted	1.83 x 2.44	VSP	7	Chenango gravelly silt loam
4	C	Portland, NY	Own-rooted	1.83 x 2.44	VSP	16	Chenango gravelly loam
4	LR	Portland, NY	Own-rooted	1.83 x 2.44	VSP	16	Chenango gravelly loam
5	C	Branchport, NY	101-14 Mgt	1.83 x 2.44	HWC	7	Valois gravelly silt loam
5	LR	Branchport, NY	101-14 Mgt	1.83 x 2.44	HWC	7	Valois gravelly silt loam
5	C	Branchport, NY	101-14 Mgt	1.83 x 2.44	VSP	14	Langford-Erie channery silt loam
5	LR	Branchport, NY	101-14 Mgt	1.83 x 2.44	VSP	14	Langford-Erie channery silt loam
6	C	Geneva-RS, NY^e^	Own-rooted	2.70 x 3.60	HWC	9	Honeoye loam
6	LR	Geneva-RS, NY	Own-rooted	2.70 x 3.60	HWC	9	Honeoye loam
6	C	Geneva-RS, NY	Own-rooted	2.70 x 3.60	VSP	9	Honeoye loam
6	LR	Geneva-RS, NY	Own-rooted	2.70 x 3.60	VSP	9	Honeoye loam
7	C	Geneva-CN, NY^f^	101-14 Mgt	2.70 x 3.60	HWC	10	Honeoye loam
7	LR	Geneva-CN, NY	101-14 Mgt	2.70 x 3.60	HWC	10	Honeoye loam

a C, Control; LR, Fruiting zone leaf removal.

b HWC, High-wire cordon; VSP, Vertical shoot-positioned system.

c Vineyard age determined as number of years from planting to the beginning of the study (2016).

d Data sourced from the USDA National Resources Conservation Service (NRCS) Web Soil Survey, https://websoilsurvey.sc.egov.usda.gov.

e Vineyard located at Cornell University AgriTech Research South (RS) farm.

f Vineyard located at Cornell University AgriTech Crittenden (CN) farm.

At each vineyard, two panels (i.e., two sections of two-post spaces) of three or four contiguous vines (1.83–2.70 m long row each) were selected for data collection. The two experimental units were randomly assigned to either a control (C; fruiting zone non-defoliated) or fruiting zone leaf removal treatment (LR). Fruiting zone leaf removal was used to maximize the range of temperatures and cluster sun exposure across sites to better assess relationships between rotundone concentration and these micrometeorological factors, rather than assess differences between C and LR treatments, as the treatments were not replicated at any site. Fruiting zone defoliation was imposed pre-veraison at Eichhorn-Lorenz (E-L) phenological stage 31, defined as “berry pea-size stage” (Coombe, 1995). Leaves were removed from each shoot within the fruiting zone, from the basal node to that above the distal cluster. Leaves were removed multiple times during both seasons to avoid vegetative re-growth in the fruiting zone. Fruiting zone defoliation was implemented on the same experimental vines during the 2016 and 2017 seasons.

### Site-Specific Weather Conditions

Vineyard air temperature, rainfall, and photosynthetically active radiation (PAR) were recorded at 15-min intervals with HOBO^®^ weather sensors and dataloggers (Onset Computer Corporation, Bourne, MA) at sites 1, 2, 3, and 4, starting on June 23 and ending on October 31 in 2016, and starting on May 1 and ending on October 31 in 2017. In 2016, weather data from May 1 until the installation of the weather stations were sourced from the nearest Network for Environment and Weather Applications (NEWA) weather station if possible (http://newa.cornell.edu). Weather data for sites 5, 6, and 7 were obtained from NEWA weather stations for both growing seasons; a NEWA weather station was located at site 5 and within 0.71 and 1.57 km from sites 6 and 7, respectively.

As HOBO^®^ weather stations only measured solar radiation from 400 to 700 nm (PAR), linear regression was used to develop a model to estimate solar radiation from PAR measurements. Briefly, a wide-spectrum (measuring a total wavelength range of 300-1100 nm) silicon pyranometer was added to the HOBO^®^ weather station at site 1 to record solar radiation and PAR concurrently. The 30-min average PAR was linearly related to solar radiation (y = 0.5099*x* – 0.0302; *r* ^2^= 0.96; *n* = 477) and used to convert PAR values to solar radiation (µmol/m^2^/s to MJ/m^2^) for the four HOBO^®^ weather stations. Concurrently, NEWA-sourced solar radiation data was converted from Langley units to MJ/m^2^ to have comparable values across all sites.

Several mesoclimatic (i.e., site-specific) parameters were calculated for each site (**Table 2**). Growing degree days (GDD), a widely used index of heat accumulation, were calculated using 10°C as a baseline (GDD = [(maximum temperature + minimum temperature)/2] – 10). Total cumulative solar exposure (CSE) was calculated as the sum of hourly solar radiation averages (MJ/m^2^). Cumulative GDD and CSE were calculated for the whole growing season (May 1 to harvest) and for the fruit ripening period (veraison-to-harvest; GDD*_v_* and CSE*_v_*) for each site (Zhang et al., 2015a).

**Table 2:** Vine and climate measurements recorded at seven Noiret vineyards during 2016 and 2017 to predict rotundone concentration in the fruit at harvest.

Vine Metrics		Climate	
Production Metrics	Nutrient and Water Status^a^	Mesoclimate^b^	Microclimate^c^
Yield	Nitrogen	Temperature	Air temperature
Cluster number	Phosphorous	GDD	Berry temperature
Cluster weight	Potassium	GDD*_v_*	CEFA
Berry weight	Magnesium	Rainfall	LEFA
Pruning weight	Calcium	Rainfall*_v_*	DH_10_
Crop load	Berry δ^13^C	Solar radiation	DH_15_
Juice soluble solids		CSE	DH_20_
Juice pH		CSE*_v_*	DH_25_
Juice titratable acidity			DH_30_
			DH_35_
			DH_40_

a Berry δ^13^C, Ratio of ^13^C:^12^C measured in grape berries at harvest.

b GDD, Seasonal growing degree days; GDD_v_, Veraison-to-harvest growing degree days; Rainfall_v_, Veraison-to-harvest rainfall; CSE, Seasonal cumulative solar exposure (MJ/m^2^)^;^ CSE_v_, Veraison-to-harvest cumulative solar exposure (MJ/m^2^).

c CEFA, Cluster exposure flux availability; LEFA, Leaf exposure flux availability; Degree-hour (DH) indexes calculated as the percentage of hours the fruit temperature was within pre-defined intervals from veraison to harvest. Temperature ranges included 10–15°C (DH_10_), 15.1–20°C (DH_15_), 20.1–25°C (DH_20_), 25.1–30°C (DH_25_), 30.1–35°C (DH_30_), 35.1–40°C (DH_35_), and >40.00°C (DH_40_).

### Fruiting Zone Weather Conditions

At each site wireless temperature data loggers (iButton Fob, Model DS9093Fl, Embedded Data Systems, Lawrenceburg, KY) were used to record air temperature at 20-min intervals in the fruiting zone of C and LR vines throughout the 2016 and 2017 seasons. Two sensors were placed within each experimental unit at the trellis wire closest to the fruiting zone, and data were averaged by experimental unit. Berry temperature was measured during fruit ripening at site 1 from September 16 to October 5, 2017, on two randomly chosen clusters from each experimental unit. For each cluster, five 12.7 mm hypodermic thermocouple probes (Model HYP1-30-1/2-T-G-60-SMP-M, Omega Engineering, Stamford, CT) were inserted into a berry at different locations within a cluster (top-east, top-west, mid-west, bottom-east, bottom-west). All 20 thermocouples were connected to a data logger unit (CR6, Campbell Scientific, Logan, UT) and berry temperatures were continuously measured and logged at 20-min intervals. Linear regression was used to fit berry flesh temperature data to the air temperature data for both the LR and C treatments (LR: y = 1.2034*x* – 2.4302, *r* ^2^ = 0.98, *n* = 96; CON: y = 0.6802*x* + 2.3627, *r* ^2^ = 0.98, n = 96). The regression equations were used to estimate berry temperature for all sites for both seasons.

Berry temperatures were used to calculate degree hour (DH) indexes, defined here as the percentage of hours the fruit temperature was within pre-defined intervals from veraison to harvest (Zhang et al., 2015a). Temperature intervals analyzed in this study were: 10–15°C, 15.1–20°C, 20.1–25°C, 25.1–30°C, 30.1–35°C, 35.1–40°C, and >40.00°C (DH_10_, DH_15_, DH_20_, DH_25_, DH_30_, DH_35_, and DH_40_, respectively). Each DH index was calculated as:

(1) $$DH_{x}=\left(\frac{\mathrm{number}\;\mathrm{of}\;\mathrm{hours}\;\mathrm{between}\;T_{1}\;\mathrm{and}\;T_{2}\;\mathrm{from}\;\mathrm{veraison}\;\mathrm{to}\;\mathrm{harvest}}{\mathrm{number}\;\mathrm{of}\;\mathrm{total}\;\mathrm{hours}\;\mathrm{between}\;\mathrm{veraison}\;\mathrm{and}\;\mathrm{harvest}}\right)*100$$

where *x* is the base temperature of the DH range, *T*_1_ is the lower threshold temperature, and *T*_2_ was the upper threshold temperature. Due to a large period of missing data, it was not possible to calculate DH indexes for sites 1, 2, 3, 4, and 5 in 2016. Values were calculated for sites 6 and 7 in 2016, and all sites in 2017.

Enhanced point quadrat analysis (EPQA; Meyers and Vanden Heuvel, 2008) was performed three times per season per site to assess canopy density and fruiting zone sunlight penetration. Each year, EPQA was measured when leaves were removed for the first time (E-L 31, “berry pea-size stage”), again at 50% veraison (E-L 35, “veraison”), and during fruit ripening between E-L 36 “berries with intermediate Brix values” and E-L 37 “berries not quite ripe” stages. Point Quadrat Analysis (PQA) was performed by inserting a thin metal rod into the grapevine fruiting zone at 20 cm intervals perpendicular to the vine row for a total of 36 insertion points per experimental unit (Smart and Robinson, 1991). PQA analysis was coupled with PAR measured within 2 h of solar noon on the same day, given full-sun conditions, using a LI-250A quantum ceptometer (LI-COR Bioscience, Lincoln, NE). Within-canopy PAR values were divided by ambient values to calculate the ratio of PAR penetrating the canopy.

Characteristics related to canopy density and fruit sunlight exposure were then analyzed using Canopy Exposure Mapping Tools (v. 1.7, freeware from J.M. Meyers, Cornell University, Ithaca, NY; Meyers and Vanden Heuvel, 2008). The software was used to calculate leaf and cluster flux availability (LEFA and CEFA, respectively), which represents the percentage of the above-canopy photo flux that reaches a leaf or cluster, respectively.

### Vine Characteristics

Harvest dates were determined by each commercial grower cooperator at sites 1, 2, 3, 4, and 5, while harvest dates at sites 6 and 7 were determined by berry sampling and assessment of fruit maturity. At harvest, total number of clusters per experimental unit was counted and weighed. Twenty clusters were randomly collected from each experimental unit, stored at -20°C, and later used for berry weight, chemical composition, carbon isotopic composition, and rotundone quantification analyses. Dormant pruning weight was measured between February and March 2017 and 2018 as the mass of 1-year-old stems (canes) produced during the preceding growing season. All yield and pruning weights were measured using a hanging scale with a 0.01 kg accuracy (Pelouze 7710, Rubbermaid, Inc., Huntersville, NC). Crop load (fruit vs. vegetative biomass) was calculated as yield divided by pruning weight (Ravaz index). In 2016, an early commercial harvest at site 5 resulted in loss of yield and related data for the experimental VSP-trained vines. Additionally, dormant pruning data were lost for 2017 at site 2 due to mixing of pruned canes between C and LR vines.

Grapevine nutrient status was determined by leaf petiole analysis at veraison 2016 and 2017 (Wolf, 2008). Thirty leaf petioles were randomly collected from each experimental unit and dried at 60°C for 48 h. Tissue samples were submitted to The Pennsylvania State University Agricultural Analytical Services Laboratory for macronutrient (N, P, K, Mg, Ca, S) and micronutrient (Mn, Fe, Cu, B, Zn) analyses by acid digestion and ICP elemental analysis (Huang and Schulte, 1985).

A 200-berry sample was randomly taken from the frozen clusters collected at harvest for each experimental unit to assess vine water status via carbon isotope composition (δ^13^C) analysis (Gaudillère et al., 2002). The 200-berry sample was split into two subsamples, oven-dried for 6 days at 60°C, frozen with N_2_ gas, ground into a powder, and submitted to the Cornell University Stable Isotope Laboratory for EA-IRMS analysis. The results were expressed as ‰ δ^13^C, or the difference in carbon isotope composition of the grape sample relative to that of the Pee Dee Belemnite internal standard. Carbon isotope composition was calculated as:

(2) $$\delta^{13}C=\left[\frac{\left(R_{g}-R_{\mathrm{pdb}}\right)}{R_{\mathrm{pdb}}}\right]*1000$$

where R_g_ = ^13^C/^12^C ratio of the grape sample and R_pdb_ = ^13^C/^12^C ratio of the Pee Dee Belemnite standard.

### Fruit Chemistry and Rotundone Analysis

In both years and for each experimental unit, fruit chemical composition data [total soluble solids (TSS), pH, and titratable acidity (TA)] were measured on a randomly selected 100-berry sample selected from the frozen clusters collected at harvest. Frozen berry samples were thawed within a plastic zip-lock bag that was heated in a water bath at 60°C, and berries were hand-crushed for juice analysis. Total soluble solids were measured using a hand-held refractometer (Master, Atago USA, Inc., Bellevue, WA) and juice pH was measured using a benchtop pH-meter (Orion Star A111, Thermo Fisher Scientific, Waltham, MA). Titratable acidity was assessed using an autotitrator (G20, Mettler Toledo, Columbus, OH) on a 10 ml juice sample titrated to an endpoint pH of 8.2 with a 0.1 M NaOH solution. Average berry weight was calculated using a 200-berry sample taken from the frozen harvested clusters.

Berry processing for rotundone extraction and analysis followed the protocol used by Homich et al. (2017). Analysis of rotundone was conducted via solid phase microextraction multidimensional gas chromatography–mass spectrometry (SPME-MDGC-MS) at the Australian Wine Research Institute (AWRI, Glen Osmond, SA) using the equipment and protocols outlined in Geffroy et al. (2014).

### Statistical Analysis

Data analysis was performed using SAS statistical software (v. 9.4, SAS Institute, Cary, NC). Relationships between all measured variables (**Table 2**) were evaluated visually using PROC GPLOT, and PROC CORR was used to assess linear correlations between rotundone concentration and the 21 variables presented in **Tables S1**, **S2**, **S3**, **S4**, and **S5**. PROC REG was used to develop a series of multiple linear regression models and identify a subset of variables to be used for a predictive model. Models were first constructed using three selection options, including FORWARD selection (α=0.1), BACKWARD elimination (α=0.1), and STEPWISE selection (α=0.1). The resulting models were compared, considering the coefficient of determination (*r* ^2^), the adjusted *r* ^2^, Mallow’s conceptual predictive criterion (*C*_p_), and mean square error (MSE; Freund and Littell, 2006).

The RSQUARE option in PROC REG was used to request all possible regressions, and all possible combinations of variables were evaluated using *r* ^2^, adjusted *r* ^2^, *C*_p_, mean square error (MSE), Bayesian information criterion (BIC), and Akaike information criterion (AIC; Freund and Littell, 2006). Candidate models were selected to evaluate model diagnostics with the *R*, INFLUENCE, VIF, and COLLINOINT options in PROC REG. Using this method, a parsimonious final predictive model (i.e., a predictive model with high explanation and the fewest necessary variables) was selected for predicting rotundone concentration in Noiret grapes.

The same statistical approach was used to identify the fruiting zone weather variables listed in **Table 2** that had the greatest influence on rotundone concentrations at harvest. Results from this regression analysis are not intended for predictive use, but for determining which micrometeorological conditions (e.g., continuous fruiting zone berry temperature or fruiting zone sun exposure measured three times) had the strongest influence on rotundone concentrations.

## Results

### Site-Specific Weather Conditions

The 2016 growing season was warmer than the 2017 season at all sites, and drier at all sites except sites 3 and 4 (**Table S1**). Seasonal heat accumulated from May 1 to the day of grape harvest was higher in 2016 than in 2017 for all seven sites. However, GDD from veraison to harvest (i.e., GDD*_v_*) were higher in 2017 as compared to 2016 in six out of the seven sites (**Table S1**). Similarly, the veraison-to-harvest period was sunnier in 2017 than in 2016 except for sites 1 and 2 (**Table S1**). Cumulative rainfall was higher in 2017 for all sites except for sites 3 and 4 (**Table S1**); the sites within the Finger Lakes AVA region (sites 5, 6, and 7) had the lowest rainfall in 2016 and the highest in 2017.

### Fruiting Zone Weather Conditions

Berry temperature between veraison and harvest was mainly within the 15.1 to 20°C range (DH_15_) at all sites except for the LR unit at site 6 (HWC) in 2016, C at site 7 in 2016, and LR at site 4 in 2017 (**Table S2**). Overall, berries were above 30 °C for a limited time during the ripening period: DH_30_ was below 5% for the C and 10% for the LR experimental units, while DH_35_ was below 1% and 5% for all the C and LR units, respectively. DH_40_ was negligible or below 1% across all sites. All C experimental units were cooler than the LR counterparts at each site, as they had higher DH_10_ and DH_15_ and lower DH_25_, DH_30_, DH_35_, and DH_40_ for all the units that have data. As expected, the percentage of ambient photon flux intercepted by both clusters (CEFA) and leaves (LEFA) was greater for the LR units as compared to the C for all sampling dates and both years (**Table S3**).

### Viticultural Data and Rotundone Concentrations

Mean values for yield parameters, pruning weight, crop load, and basic juice chemistry are reported in **Table S4**. As expected, there was large variation in production parameters which was, at least in part, explained by the different management practices (i.e., shoot and cluster thinning) used by the grower cooperators. Yield, for example, varied overall between 1.54 (site 2 LR) and 6.72 (site 5 C) kg/m of cordon. Basic juice chemistry (TSS, pH, TA) values were within the range of those reported for Noiret in previous studies conducted in the northeast U.S. (Vanden Heuvel et al., 2013; Homich et al., 2017).

Concentrations of the major macronutrients were at deficiency levels for some of the sites, although visual symptoms of leaf nutrient deficiency were not observed except for Mg (**Table S5**). For example, concentration of leaf petiole N was at deficiency level (<0.80%) for a few experimental units in 2016 (site 3 C; site 6) and for more sites in 2017 (site 2; site 4; site 6 LR HWC and VSP units; and site 7 LR). Phosphorus concentration for site 7 (2016, 2017) and for the C unit at site 3 (2016) was in the deficiency range (<0.14%), while K concentration was low (<1.20%) for sites 2 and 6 in 2017.

Conversely, there were two sites in 2016 (site 2; site 6 C) and more in 2017 (site 2; site 4; site 6) that exceeded the recommended late-season P leaf petiole concentration (0.14–0.30%). Potassium exceeded recommended concentrations (1.2–2.0%) at site 1 in both years, while at site 5 three out of the four experimental units had excessive concentration of K in 2016 (HWC C and VSP) and 2017 (HWC C and VSP). Likely because of excessive K uptake, Mg concentration at site 1 in both 2016 and 2017 was at a deficiency level (<0.35%), and visual Mg deficiency symptoms were observed in both seasons.

Berry carbon isotope ratio, a proxy for vine water status, exhibited moderate variation between years (**Table S5**). On average, δ^13^C ranged from -24.8, i.e., lower water status (site 7 LR), to -29.4, i.e., higher water status (site 2 C), in 2016, and from -27.1 (Site 5 C) to -29.7 (site 6 C) in 2017. Inter-annual variability was high at sites 6 and 7, while δ^13^C values at sites 1 and 2 remained nearly consistent across both years.

**Figure 2:**
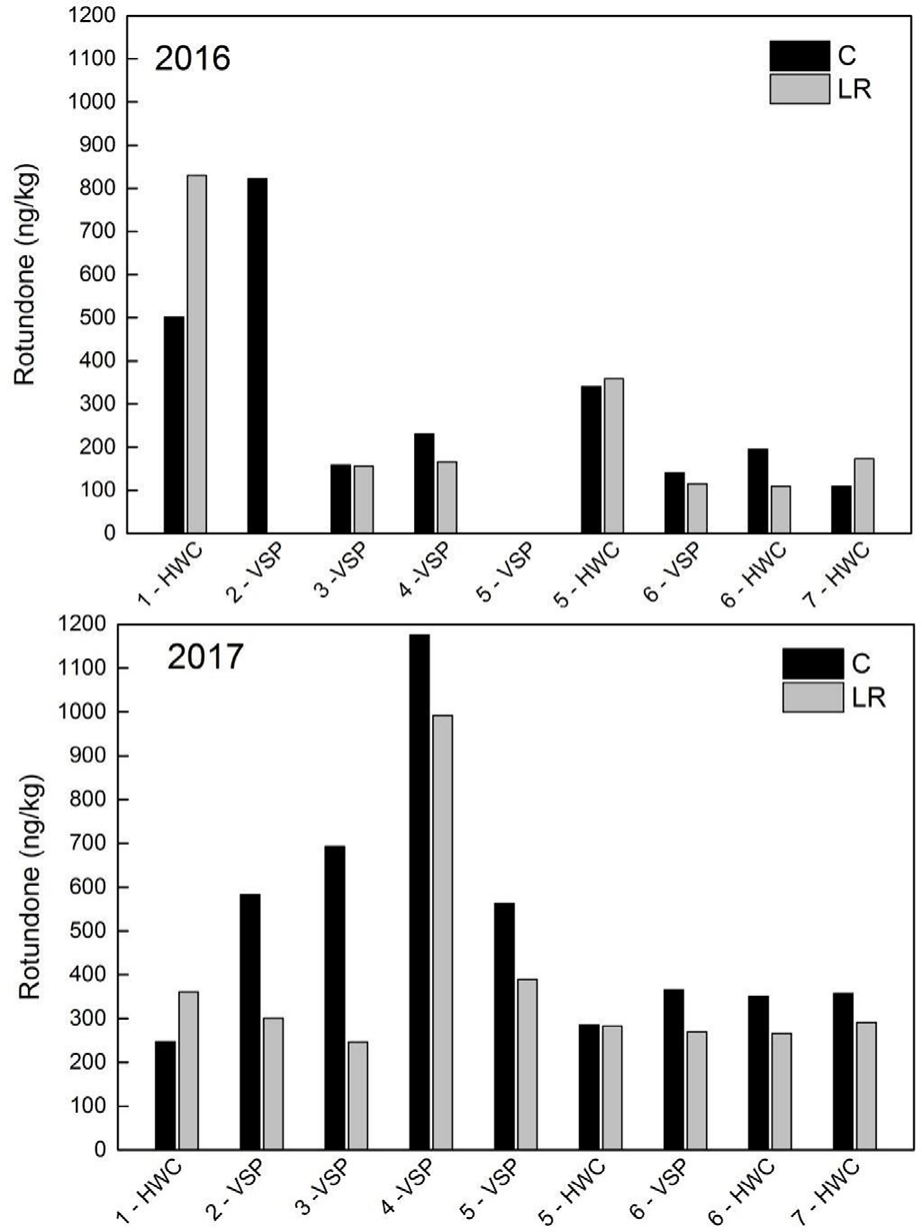
Berry rotundone concentrations at harvest in 2016 and 2017 for each Noiret site for control (C; black bars) and fruiting zone leaf removal (LR; gray bars), HWC, High-wire cordon; VSP, Vertical shoot-positioned system. Bars indicate one value for site; experimental units were not replicated within the site.

Berry rotundone concentration at harvest exhibited both inter-site and inter-annual variation (**Figure 2**). Values ranged from 108.9 ng/kg (site 5 LR) to 830.2 ng/kg (site 1 LR) in 2016 and from 246.6 ng/kg (site 3 LR) to 1176.1 ng/kg (site 4 C) in 2017. The LR unit at site 2 was omitted from the analysis due to issues with sample analysis. Sites in the Finger Lakes AVA displayed moderate to high variation between years in rotundone concentration for both treatments, while rotundone concentration at site 4 was on average more than four times higher in 2017 when compared to the previous vintage (**Figure 2**). Conversely, site 1 experienced the highest decrease in rotundone concentration from the first to the second year, with 2017 concentration being less than half that of 2016. All C units except for site 1 tended to have higher rotundone concentrations, between 0.7% and 64.4%, than the respective LR units in 2017. Trends were less consistent in 2016: in addition to site 1, rotundone tended to be higher for LR units at site 5 HWC (5.2%) and site 7 (18.6%) as compared to the C.

### Multiple Linear Regression Analysis and Selection of a Rotundone Mesoclimatic Model

Scatter plots indicated that rotundone concentration was linearly related to K, Mg, Ca, average berry weight, average cluster weight, GDD, GDD*_v_*, and rainfall, and both linearly and quadratically related to CSE and CSE*_v_* (data not shown). Pearson correlation coefficients (*r*) were used to assess the strength of linear correlations for both pooled and yearly data (**Table 3**).

**Table 3:** Pearson correlation coefficient representing the linear relationships between rotundone, vine production, vine water and nutrient status, mesoclimate, and microclimate parameters measured at each of the seven Noiret vineyards in 2016 and 2017. Correlation coefficients were measured for both yearly (2016 and 2017) and pooled (2016 & 2017) data. Bolded font indicates a significant relationship (*p* < 0.05).

Variable	Rotundone			Variable	Rotundone		
	2016	2017	2016 & 2017		2016	2017	2016 & 2017
*Production*				*Site weather*^*a*^			
TSS	**–0.69**	–0.21	**–0.48**	GDD	–0.37	**0.50**	–0.11
pH	-0.03	0.42	0.12	Rainfall	0.46	**–0.52**	0.09
TA	0.24	–0.46	0.11	CSE	0.37	0.38	**0.42**
Berry wt	0.46	**0.72**	**0.56**	GDD_v_	**0.53**	**0.75**	**0.70**
Cluster wt	0.46	0.21	0.22	Rainfall_v_	–0.15	**0.72**	0.33
Cluster no.	–0.40	0.12	–0.01	CSE_v_	0.75	0.73	0.74
Yield	–0.19	0.17	0.07				
Pruning wt	0.23	–0.22	0.02				
Crop load	–0.20	0.12	0.04				
*Vine water and nutrient status*				*Fruiting zone weather*^*b*^			
δ^13^C	**–0.70**	0.39	–0.33	DH_10_	0.13	–0.14	–0.30
N	**0.56**	–0.16	0.11	DH_15_	0.29	0.27	**0.40**
P	0.28	0.07	0.23	DH_20_	–0.21	**0.58**	0.35
K	0.47	0.20	0.28	DH_25_	–0.03	0.00	–0.05
Mg	**–0.56**	–0.41	**–0.50**	DH_30_	–0.02	–0.23	–0.28
Ca	**–0.84**	–0.10	**–0.44**	DH_35_	–0.10	–0.25	–0.30
				DH_40_	–0.19	–0.15	–0.27
				LEFA*_p_*	–0.09	0.12	–0.07
				LEFA*_v_*	0.04	0.12	0.04
				LEFA*_r_*	–0.17	–0.05	–0.08
				CEFA*_p_*	–0.24	0.07	–0.07
				CEFA*_v_*	0.00	0.03	–0.01
				CEFA*_r_*	–0.21	–0.08	–0.11

a GDD, Seasonal growing degree days; GDD_v_, Veraison-to-harvest growing degree days; Rainfall_v_, Veraison-to-harvest rainfall; CSE, Seasonal cumulative solar exposure (MJ/m^2^); CSE_v_, Veraison-to-harvest cumulative solar exposure (MJ/m^2^).

b DH_x_, Percent of degree-hours between 10.1–15°C (DH_10_), 15.1–20°C (DH_15_), 20.1–25°C (DH_20_), 25.1–30°C, 30.1–35°C (DH_30_), 35.1–40°C (DH_35_), and > 40°C (DH_40_); LEFA and CEFA, Leaf and cluster exposure flux availability, measured at berry pea-size stage (p), veraison (v), and during grape ripening (r).

The production variables most strongly correlated with rotundone in 2016 and for 2016 and 2017 combined were berry weight and TSS. For the remaining production variables, except for cluster weight, the relationships were inconsistent between the two years and altogether poorly correlated with rotundone concentration. The relationship between δ^13^C and rotundone concentration was negative in 2016 and positive in 2017. Among leaf petiole nutrients, both Mg and Ca showed the highest, negative correlations with rotundone.

Weather parameters were better correlated with rotundone when measured from veraison to harvest instead of for the whole growing season (**Table 3**). Specifically, CSE*_v_* exhibited the highest positive correlation with rotundone when data from the two years were combined (*r* = 0.74, *p* < 0.001), followed by GDD*_v_*. Both linear and quadratic terms were included in the regression analysis for the variables CSE and CSE*_v_*, as the quadratic terms were better correlated to rotundone concentration.

Several candidate regression models were evaluated using different selection options, but they did not provide the optimal model with any given predictor variables, as they were prone to overfitting the data (Freund and Littell, 2006). Therefore, the RSQUARE option was used with PROC REG to better fit the data and aid in model selection. The best three models out of all models generated for one-, two-, three-, four-, five-, and six-variable models with the RSQUARE option are reported in **Table 4**, including values for various statistical parameters used for model selection. Analysis of *r* ^2^, adjusted *r* ^2^, *C*_p_, AIC, BIC, and MSE values suggested that a six-variable model may be overfitted (Freund and Littell, 2006) and that a lower-variable model may be better-suited for predictive purposes (**Table 4**).

**Table 4:** The best multi-variable models for rotundone prediction for one to six regressor variables, along with fit statistics, using mesoclimate, vine production, and physiological data from seven Noiret vineyards over two years (2016 and 2017).The four-variable model shown at the bottom of the table emerged as the strongest candidate for use as a predictive model (*n* = 34).

No. of Variables	Model variables^a^	*r* ^2^	*C*_p_^b^	AIC^c^	BIC^d^	MSE^e^
1	CSE*_v_* ^2^	0.670	15.7	297.0	297.7	26295
1	CSE*_v_*	0.585	26.2	303.7	303.5	33059
1	GDD*_v_*	0.512	35.3	308.4	307.8	38902
2	CSE, CSE_v_^2^	0.792	2.62	285.5	288.3	17161
2	CSE, CSE_v_	0.772	5.12	288.2	290.4	18830
2	CSE_v_^2^, CSE_v_	0.741	8.93	291.9	293.4	21385
3	CSE_v_^2^, CSE, crop load	0.830	–0.05	281.7	286.5	14582
3	CSE_v_^2^, CSE, pruning wt	0.817	1.63	283.9	288.0	15760
3	CSE_v_^2^, CSE, GDD_v_	0.816	1.77	284.1	288.2	15858
4	CSE_v_^2^, CSE_v_, GDD_v_, crop load	0.858	–1.45	278.5	286.2	12724
4	CSE_v_^2^, CSE_v_, crop load, Ca	0.856	–1.27	278.9	286.4	12858
4	GDD_v_, crop load, Ca, P	0.853	–0.85	279.5	286.8	13162
5	CSE_v_^2^, CSE_v_, crop load, Ca,	0.885	–2.81	274.4	286.7	10739
5	CSE_v_^2^, CSE_v_, crop load, Ca, pH	0.882	–2.49	275.0	287.0	10976
5	CSE_v_^2^, CSE, crop load, Ca, N	0.877	–1.80	276.6	287.6	11502
6	CSE_v_^2^, CSE_v_, Ca, pruning wt, rain, yield	0.899	–2.59	272.5	289.6	9813
6	CSE_v_^2^, CSE_v_, Ca, pruning wt, rain, cluster no.	0.899	–2.53	272.6	289.7	9860
6	Ca, pruning wt, rain, cluster no., P, GDD_v_	0.897	–2.35	273.1	289.8	10004
Best regression model equation to be used for rotundone prediction						
Year	Model				*r* ^2^	Adj. *r* ^2^
2016 & 2017	Rot. = –530.4 + 568.4 * P – 336.4 * Ca + 18.4 * crop load + 3.9 *GDD*_v_*				0.853	0.828

^a^GDD, Seasonal growing degree days; GDD_v_, Veraison-to-harvest growing degree days; Rainfall_v_, Veraison-to-harvest rainfall; CSE, Seasonal cumulative solar exposure (MJ/m^2^); CSE_v_, Veraison-to-harvest cumulative solar exposure (MJ/m^2^).^b^C_p_, Mallow’s C_p_ statistic; ^c^AIC, Akaike information criterion; ^d^BIC, Bayesian information criterion; ^e^MSE, Mean square error.

As more variables were added to the models, less additional variation was explained by each additional variable; this is reflected in the 0.028 increase in *r* ^2^ when a fourth variable is added to the model, for example, when compared to the 0.027 increase when a fifth variable is added (**Table 4**). A slight decrease in MSE between the best fourth- and fifth-variable model indicated that each new variable added again explained a diminishing proportion of variation, and that models with fewer variables may be better suited for predictive purposes. Based on this, while also considering multicollinearity diagnostic statistics, *F*-values, adjusted *r* ^2^ values, and other model-selection statistical criteria, the four-variable model including GDD*_V_*, Ca, crop load, and P emerged as the strongest candidate for use as a predictive model. Compared to other candidate models analyzed, this four-variable model was the best due to its increased predictive power (**Table 4**).

### Partial Validation of Predictive Rotundone Mesoclimatic Model

**Figure 3:**
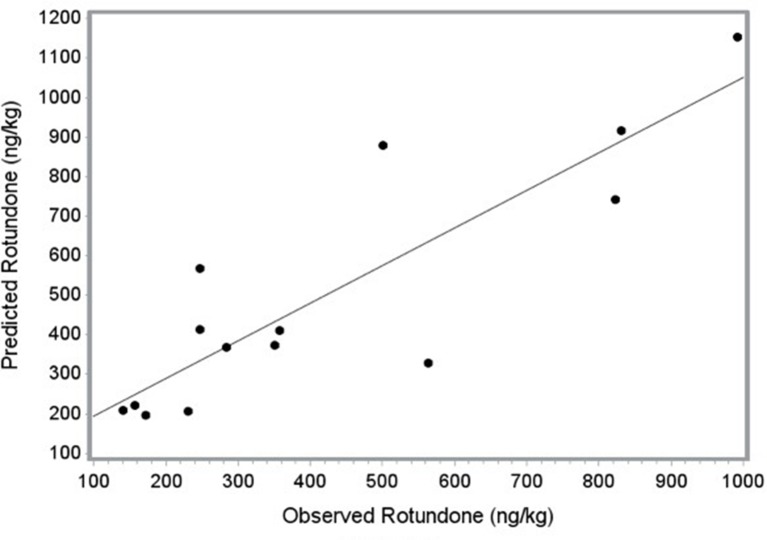
Relationship between predicted and observed rotundone concentrations (ng/kg) generated using SAS’ PROC SCORE. Regression equation for the two-year data set (n = 14): Rotundone (ng/kg) = –530.4 + 568.4 * P – 336.4 * Ca + 18.4 * crop load + 3.9 *GDD_v_, where P and Ca are phosphorus and calcium concentrations in the leaf petiole, respectively, and GDD_v_ are the growing degree days accumulated from veraison to harvest. Partial validation regression equation: Predicted rotundone (ng/kg) = 98.1 + 0.95*Observed Rotundone; *r* ^2^ = 0.753; *p* < 0.05.

To validate the strength of the chosen model, the dataset was split into two randomized datasets (*n* = 20, *n* = 14) and multiple linear regression was performed on the first validation data subset (Freund and Littell, 2006). The same four-variable model (GDD*_v_*, Ca, crop load, and P) was selected by FORWARD selection and the RSQUARE option as the optimal fit for the validation data subset. The model equation was then used to generate predicted rotundone concentrations for the second validation data subset (*n* = 14). The strong linear relationship between predicted and observed rotundone concentrations support the use of the four-variable model as a predictive model for determining rotundone concentrations (Freund and Littell, 2006; **Figure 3**).

### Multiple Linear Regression Analysis and Selection of a Rotundone Microclimatic Model

Rotundone concentration was poorly correlated with berry temperature (**Table 3**). Scatter plots indicated weak negative linear relationships between rotundone concentration and all DH indices except for DH_15_ and DH_20_, which were positively correlated with rotundone for the 2-year dataset. Pearson correlation coefficients supported these visual interpretations, as the *r* values for all DH indices were low and relationships were nonsignificant except for DH_15_ (DH_10_: *p* = 0.14; DH_15_: *p* = 0.04; DH_20_: *p* = 0.08; DH_25_: *p* = 0.80; DH_30_: *p* = 0.17; DH_35_: *p* = 0.15).

There was not a clear visual linear trend between rotundone concentration and the percentage of sunlight reaching the leaves (LEFA) or the clusters (CEFA) in the fruiting zone for any of the three sampling dates (data not shown). Rotundone concentration was, indeed, poorly correlated with the LEFA and CEFA in both years (**Table 3**).

Different candidate models were evaluated and results indicated that a three-variable model (DH_10_, DH_30_, and CEFA*_p_*) was the best candidate with an *r* ^2^ of 0.57 and an adjusted *r* ^2^ of 0.51 (**Table 5**). Further diagnostic analyses of multicollinearity, model residuals, outliers, and influential observations reaffirmed the strength of the three-variable model as the best candidate model.

**Table 5:** The best multi-variable models explaining microclimatic influence on rotundone for one to six regressor variables, along with fit statistics, using microclimate data from seven Noiret vineyards over two years (2016 and 2017). The three-variable model shown at the bottom of the table emerged as the strongest candidate for use as a predictive model (*n* = 24).

No. of Variables	Model variables^a^	*r* ^2^	*C*_p_^b^	AIC^c^	BIC^d^	MSE^e^
1	DH_15_	0.164	4.50	266.6	268.5	61721
1	DH_20_	0.122	5.58	267.6	269.4	64425
1	DH_10_	0.096	6.51	268.5	270.1	66770
2	DH_30_, CEFA*_p_*	0.494	–3.16	256.5	261.5	39132
2	DH_30_, DH_10_	0.407	–0.63	260.3	264.3	45815
2	DH_15_, CEFA*_p_*	0.363	0.65	262.0	265.6	49222
3	DH_30_, CEFA*_p_*, DH_10_	0.574	–3.53	254.4	262.1	34534
3	DH_30_, CEFA*_p_*, DH_15_	0.543	–2.61	256.1	263.2	37077
3	DH_30_, CEFA*_p_*, CEFA*_r_*	0.536	–2.41	256.4	263.4	37621
4	DH_30_, CEFA*_p_*, DH_10_, DH_35_	0587	–1.89	255.7	265.6	35302
4	DH_30_, CEFA*_p_*, DH_10_, DH_25_	0.584	–1.80	255.8	265.6	35556
4	DH_30_, CEFA*_p_*, DH_10_, DH_20_	0.582	–1.74	256.0	265.7	35744
5	DH_30_, CEFA*_p_*, DH_10_, DH_20_, DH_25_	0.606	–0.44	256.5	269.1	35556
5	DH_30_, CEFA*_p_*, DH_10_, LEFA*_p_*, DH_35_	0.598	–0.22	257.0	269.3	36240
5	DH_30_, CEFA*_p_*, DH_10_, CEFA*_v_*, DH_35_	0.597	–0.19	257.1	269.3	36346
6	DH_30_, CEFA*_p_*, DH_10_, DH_25_, CEFA*_v_*, DH_35_	0.610	1.41	258.3	273.3	37206
6	DH_30_, CEFA*_p_*, DH_10_, DH_25_, CEFA*_v_*, DH_20_	0.610	1.42	258.3	273.3	37228
6	DH_30_, CEFA*_p_*, DH_10_, DH_25_, DH_20_, DH_35_	0.609	1.46	258.4	273.3	37362
Best regression model equation for explaining microclimatic influence on rotundone						
Year	Model				*r* ^2^	Adj. *r* ^2^
2016 & 2017	Rot. = 972.5 – 21.6 * DH_10_ – 114.4 * DH_30_ + 1230.8 * CEFA*p*				0.574	0.511

^a^DH_x_, Percent of degree-hours between 10.1–15°C (DH_10_), 15.1–20°C (DH_15_), 20.1–25°C (DH_20_), 25.1–30°C, 30.1–35°C (DH_30_), 35.1–40°C (DH_35_), and >40°C (DH_40_); LEFA and CEFA, Leaf and cluster exposure flux availability, measured at berry pea-size stage (p), veraison (v), and during grape ripening (r).

^b^C_p_, Mallow’s C_p_ statistic; ^c^AIC, Akaike information criterion; ^d^BIC, Bayesian information criterion; ^e^MSE, Mean square error.

## Discussion

Various attempts have been made to understand the relationships between individual environmental or viticultural factors and rotundone concentration in grapes and wine (Geffroy et al., 2014; Zhang et al., 2015a; Zhang et al., 2015a; Homich et al., 2017); however, we are unaware of any studies that comprehensively evaluated the relationships among viticultural variables, meso- and microclimate, and rotundone concentration in grapes. Similar to previous work, in our study concentrations of rotundone in Noiret berries at harvest exhibited high variation both geographically and inter-annually (Scarlett et al., 2014; Logan, 2015; Bramley et al., 2017), indicating that our data set was a good candidate for regression analysis. Overall, rotundone concentrations ranged from 108 ng/kg (site 6 HWC LR, 2016) to 1176 ng/kg (site 4 C, 2017), falling within the middle range of those reported worldwide, including Australian Shiraz wine grapes (Wood et al., 2008; Caputi et al., 2011; Takase et al., 2015; Bramley et al., 2017).

We constructed a four-variable predictive model that explained ca. 83% of rotundone concentration variation in Noiret grapes at harvest using data from both study years. The multiple linear regression model indicated the rotundone concentration was positively related to GDD*_v_*, crop load and P, and negatively related to Ca concentration in leaf petiole. Analysis of model residuals and partial model validation supported the strength of the chosen model, though to increase confidence in the model it would be necessary to further validate it with an external data set. This would ideally be done using additional weather, nutritional, production, and rotundone data.

Our goal was to include variables that are significant for explaining rotundone concentration, but also easily measured by growers and researchers. The four variables selected (GDD*_v_*, Ca, crop load, and P) satisfy these requirements while maintaining a high degree of predictive power. Leaf petiole nutrient analysis is typically conducted annually by growers. Crop load is calculated after harvest when vines are pruned during the dormant season; however, historic crop load values could be used in the model assuming similar management practices are applied over the years and no relevant crop or vegetative tissue losses are recorded. Growers can estimate GDD*_v_* prior to harvest, as GDD accumulate slowly during the last couple of weeks prior to harvest in cool climates.

We expected GDD*_v_* to be included in the predictive model, as it was one of the variables best correlated with rotundone concentration. Weather conditions from veraison to harvest were better predictors of rotundone concentrations than season-long weather variables, reaffirming the importance of measuring those parameters when rotundone is accumulating in the berries (Zhang et al., 2015a). However, linear correlations between rotundone concentration at harvest and both GDD*_v_* and CSE*_v_* contrasted with those reported in previous work. In our cool climate region, rotundone concentrations in the fruit were highly and positively correlated to GDD*_v_* and CSE*_v_* during the ripening period, while in a previous study conducted in Australia higher GDD and solar radiation during fruit ripening negatively affected rotundone concentration in Shiraz wines (Zhang et al., 2015a).

It is unclear why results from the two studies were contradictory; reasons could potentially include the fact that we investigated a *Vitis* hybrid while Zhang et al. (2015a) studied a *Vitis vinifera* variety (Shiraz), or that we evaluated a wider range of GDD*_v_* and veraison-to-harvest solar exposure (i.e., total amount of solar radiation reaching the site in a day). Furthermore, while we evaluated seven sites for two seasons, Zhang et al. (2015a) evaluated 15 seasons of data at the same site. At the Australian site, GDD*_v_* ranged from about 300 to 408 and solar exposure during fruit ripening from 14.2 to 22.8 MJ/m^2^/day across 15 seasons. In our study, GDD*_v_* varied from 249 to 457, and solar exposure during fruit ripening from 7.6 to 21.1 MJ/m^2^/day. Specifically, some of our sites were exposed to lower solar radiation from veraison to harvest. Cluster shading imposed at various phenological stages decreased rotundone concentrations in Australian Shiraz wine grapes (Zhang, 2015), suggesting that accumulation of rotundone may be mediated by solar radiation. While it is unknown if this relationship is direct or indirect, it is possible that such low levels of solar radiation at some of our sites, and related air temperatures, were suboptimal for rotundone accumulation. Nevertheless, the significant relationship between CSE*_v_* and rotundone concentration warrants further investigations.

The length of the ripening period can also influence rotundone concentrations at harvest, which possibly contributes to explaining different results across studies. In Duras, rotundone reached peak concentrations around 44 days following veraison and thereafter decreased slightly (Geffroy et al., 2014). Here, the sites with the longest ripening periods (site 1: 47 days in 2016; site 4: 52 in 2017) had the highest rotundone concentrations, while at site 1 a shorter ripening period in 2017 compared to the previous vintage corresponded with lower rotundone concentration at harvest. This suggests that a longer ripening period in our cool climate might favor rotundone accumulation without reaching a plateau or decrease in concentration as reported in other grape growing regions where grapes are typically exposed to higher temperature or solar radiation (e.g., Australia or France).

Linear correlations between rotundone concentration and production variables were not as strong as those with weather parameters. The variables with the highest correlation coefficients were berry weight (*r* = 0.56) and TSS (*r* = - 0.48), although neither of those parameters were selected as predictor variables for the multiple linear regression model. There is no indication of a direct relationship between rotundone and TSS; it is instead possible that this relationship reflects the contrasting influence of environmental parameters like vine water status (i.e., δ^13^C) on rotundone and TSS. In 2016, TSS was positively correlated (*r* = 0.68), while rotundone was negatively correlated (*r* = - 0.70) with δ^13^C. Likewise, it is possible that environmental factors such as vine water status and heat accumulation affected berry weight (δ^13^C: *r* = - 0.72, *p* < 0.001 in 2016; GDD*_v_* *r* = 0.55, *p* < 0.001) and rotundone similarly.

Our results suggest that rotundone is more sensitive to vine water status during seasons with less precipitation, and particularly if vines may experience water deficit. Correlation between rotundone and vine water status varied by year. Carbon isotope ratio (δ^13^C) correlated negatively and significantly (*p* = 0.004) with rotundone in 2016, a relatively dry year, when δ^13^C reached values that would indicate weak-to-moderate (-26 to -25‰) and moderate-to-severe (-25 to -24‰) water deficit (Santesteban et al., 2015). Furthermore, the region where sites 5, 6, and 7 were located (i.e., the Finger Lakes AVA) experienced severe drought conditions during the 2016 season (Sweet et al., 2017), which indicates that experimental vines might have experienced water deficit during the season. Our 2016 data suggest that vines with lower water status have less rotundone in the fruit at harvest as reported in previous work (Geffroy et al., 2014). However, correlation between rotundone and δ^13^C was not significant in 2017 (*p* = 0.106), a relatively wet year when δ^13^C values were relatively similar across sites, ranging from -27.1 to -29.1.

Linear correlations between concentration of rotundone and several macronutrients (e.g., K, Mg, and Ca) in leaf petiole tissues were similar for the two years. Both Ca and P were included as predictor variables within the model. However, it is unclear if these or other nutrients had direct influence on rotundone accumulation in the berries. Instead, it is again possible that environmental conditions that led to greater plant uptake of Ca and Mg, such as decreased seasonal rainfall (Wolf, 2008), were also conducive to lower rotundone concentrations. Indeed, previous work suggested a positive relationship between seasonal rainfall and rotundone concentration (Zhang et al., 2015a). Calcium was correlated with δ^13^C in 2016 (*r* = 0.77, *p* = < 0.001), indicating that Ca concentration was positively influenced by decreasing vine water status. Conversely, P concentration in leaf petiole was positively correlated with seasonal rainfall across the two years (*r* = 0.39, *p* = 0.016). To our knowledge, our study was the first to explore and report significant relationships between rotundone concentrations and grapevine macronutrient concentrations. The inclusion of Ca and P within the final model suggests a necessity to further investigate potential direct versus indirect effects of grapevine nutrition on rotundone concentrations.

Crop load was the last variable included in the predictive model, despite the absence of a correlation with rotundone concentration when analyzed by itself. It should be remembered that the inclusion of any variable in the model is based on its relationship with all the other regressor variables in the model, and that the inclusion of multiple highly correlated, or collinear, variables will result in a model with poor predictive ability. The multiple linear regression includes predictor variables that explain a high degree of the response variable (i.e., rotundone) variation while minimizing any collinearity between predictor variables. This is why, for example, GDD*_v_* but not CSE*_v_* was included in the model despite CSE*_v_* also being highly correlated with rotundone. CSE*_v_* and GDD*_v_* are highly collinear variables and any model containing both of these variables is unfit for use as a predictive model. Crop load can be correlated with other regressor variables, not included in the model, and be the one of four best variables explaining variation in rotundone concentration without creating collinearity issues.

Regression analysis among variables measured at the fruiting zone level included DH_10_, DH_30_, and CEFA*_p_* as predictor variables. Although these three variables comprise a simple model with low predictive capabilities (51%), it provided further clarification as to which specific micrometeorological factors had the strongest influence on rotundone at the fruiting zone-scale. Overall, rotundone concentration was negatively correlated to low (<15°C) and high (>30°C) berry temperatures, and positively correlated to temperatures between 15 and 25°C, though the strength of these relationships tended to be weak. Thus, rotundone might be limited by ripening periods characterized by excessively cool or hot temperatures, as suggested by previous work (Zhang et al., 2015a). Whereas Noiret berries were exposed to cool temperatures (<15°C) for up to 30% of the fruit ripening period, berries rarely exceeded 30°C in our cool climate.

The selection of CEFA*_p_* as a predictive variable was unexpected as cluster sunlight exposure correlated poorly with rotundone across the season, from treatment application to harvest. While it is hard to decouple solar radiation and temperature effects on rotundone concentrations (Geffroy et al., 2014), previous work suggested that sunlight exclusion had a negative effect on rotundone accumulation (Zhang, 2015). Furthermore, rotundone concentrations were higher in Noiret fruit harvested from vines with higher CEFA*_p_*, when compared to non-defoliated vines, but multiple years of data are not available to confirm this relationship (Homich et al., 2017). Pre-veraison increased solar exposure might potentially affect the concentration of the aroma precursor to rotundone, the sesquiterpene α-guaiene, as suggested by Homich et al. (2017), and thus regulate rotundone concentration indirectly. Indeed, α-guaiene concentrations in Shiraz grapes were lower in the shading compared to the non-shading treatment (Zhang, 2015). Better understanding micrometeorological influence on this compound may help unravel the effects of fruiting zone solar exposure and temperature on rotundone.

Our results did not confirm a negative relationship between fruit sun exposure and rotundone concentrations as suggested by previous work, which, however, did not directly assess fruiting zone solar exposure (Scarlett et al., 2014; Zhang et al., 2015a; Zhang et al., 2015b). Differences in experimental design among studies assessing defoliation-induced influence upon rotundone make comparison difficult because treatments were imposed at different vine phenological stages (Geffroy et al., 2014; Zhang et al., 2015a; Homich et al., 2017). Furthermore, the effect of sunlight cluster exposure on rotundone concentration should be analyzed within the context of a site’s weather conditions, in that exposing fruit to increased solar radiation might affect rotundone concentration differently in a warm and sunny site as compared to a cool and cloudy site.

## Conclusion

In this study, 21 vineyard-scale regressor variables were used to develop a multiple linear regression model with high predictive power for rotundone concentration in Noiret fruit at harvest. Correlations between weather parameters and rotundone concentrations were overall weaker at the fruiting zone rather than at the site-level, although some trends were identified. The predictive model was tailored to grower application and included easily measurable variables: GDD*_v_*, Ca and P petiolar concentrations at veraison, and crop load. Although model validation is needed to verify the predictive power of the model, the strong correlations between some of the regressor variables (e.g., GDD*_v_* and CSE*_v_*) and rotundone concentration suggest that data presented here could be used by Noiret growers to identify sites more conducive to rotundone production in our cool climate.

## Data Availability Statement

All datasets generated for this study are included in the manuscript/**Supplementary Files**.

## Author Contributions

MC and JV developed the project. AH, MC, RE, RM, and JV contributed to the experimental design and data collection plans. AH led the field work. AH and RE contributed to the laboratory work. AH and RM conducted the statistical data analysis. AH and MC contributed to data interpretation and led the writing. All authors contributed to the manuscript review.

## Funding

This study was supported by the Pennsylvania Wine Marketing Research Board, the John H. and Timothy R. Crouch Program Support Endowment, and the USDA National Institute of Food and Agriculture Federal Appropriation under Project PEN0 4628 and Accession number 1014131.

## Conflict of Interest

The authors declare that the research was conducted in the absence of any commercial or financial relationships that could be construed as a potential conflict of interest.

## Supplementary Material

The Supplementary Material for this article can be found at: https://www.frontiersin.org/articles/10.3389/fpls.2019.01255/full#supplementary-material
