# Supplementary material for: Modeling the Impacts of Weather and Cultural Factors on Rotundone Concentration in Cool-Climate Noiret Wine Grapes
Source: Front Plant Sci. 2019 Oct 15;10:1255. doi: 10.3389/fpls.2019.01255 (PMC6803480; doi:10.3389/fpls.2019.01255)
Supplement: Supplementary file 1 [file Table_1.docx]

| **Supplementary Table 1**. Weather data measured for the seven Noiret vineyards during growing seasons (May 1 to October 31) and berry ripening period (veraison-to-harvest) in 2016 and 2017. | | | | | | | |
| --- | --- | --- | --- | --- | --- | --- | --- |
| **Year** | **Site** | **GDD^a^** | **GDD*_v_*^b^** | **Rainfall**  **(mm)** | **Rainfall*_v_*^c^**  **(mm)** | **CSE^d^**  **(MJ/m^2^)** | **CSE*_v_*^e^**  **(MJ/m^2^)** |
| 2016 | 1 | 1526 | 360 | 319 | 86 | 2804 | 742 |
|  | 2 | 1474 | 312 | 475 | 120 | 3271 | 730 |
|  | 3 | 1551 | 278 | 382 | 110 | 3379 | 548 |
|  | 4 | 1547 | 307 | 395 | 162 | 3217 | 581 |
|  | 5 | 1438 | 249 | 287 | 73 | 2067 | 410 |
|  | 6 | 1523 | 293 | 237 | 96 | 1737 | 306 |
|  | 7 | 1528 | 291 | 238 | 97 | 1747 | 317 |
| 2017 | 1 | 1292 | 253 | 495 | 35 | 2789 | 552 |
|  | 2 | 1417 | 341 | 512 | 81 | 3112 | 689 |
|  | 3 | 1401 | 375 | 301 | 16 | 3097 | 709 |
|  | 4 | 1468 | 457 | 384 | 197 | 3318 | 861 |
|  | 5 | 1305 | 271 | 515 | 84 | 2480 | 506 |
|  | 6 | 1405 | 320 | 635 | 79 | 3123 | 616 |
|  | 7 | 1412 | 327 | 635 | 79 | 3131 | 624 |
| ^a^GDD = Seasonal growing degree days.  ^b^GDD*_v_* = Veraison-to-harvest growing degree days.  ^c^Rainfall*_v_* = Veraison-to-harvest rainfall.  ^d^CSE = Seasonal cumulative solar exposure.  ^e^CSE*v* = Veraison-to-harvest cumulative solar exposure. | | | | | | | |
