# Supplementary material for: Modeling the Impacts of Weather and Cultural Factors on Rotundone Concentration in Cool-Climate Noiret Wine Grapes
Source: Front Plant Sci. 2019 Oct 15;10:1255. doi: 10.3389/fpls.2019.01255 (PMC6803480; doi:10.3389/fpls.2019.01255)
Supplement: Supplementary file 2 [file Table_2.docx]

| **Supplementary Table 2.** Berry temperature indexes (DH) measured during the berry ripening period (veraison-to-harvest) for 2016 and 2017. | | | | | | | | | | |
| --- | --- | --- | --- | --- | --- | --- | --- | --- | --- | --- |
| **Year** | **Site** | **Training system^a^** | **Treat-**  **ment^b^** | **DH_10_^c^ (%)** | **DH_15_ (%)** | **DH_20_ (%)** | **DH_25_**  **(%)** | **DH_30_**  **(%)** | **DH_35_**  **(%)** | **DH_40_**  **(%)** |
| 2016 | 1 | HWC | C | NA^d^ | NA | NA | NA | NA | NA | NA |
|  | 1 | HWC | LR | NA | NA | NA | NA | NA | NA | NA |
|  | 2 | VSP | C | NA | NA | NA | NA | NA | NA | NA |
|  | 2 | VSP | LR | NA | NA | NA | NA | NA | NA | NA |
|  | 3 | VSP | C | NA | NA | NA | NA | NA | NA | NA |
|  | 3 | VSP | LR | NA | NA | NA | NA | NA | NA | NA |
|  | 4 | VSP | C | NA | NA | NA | NA | NA | NA | NA |
|  | 4 | VSP | LR | NA | NA | NA | NA | NA | NA | NA |
|  | 5 | HWC | C | NA | NA | NA | NA | NA | NA | NA |
|  | 5 | HWC | LR | NA | NA | NA | NA | NA | NA | NA |
|  | 5 | VSP | C | NA | NA | NA | NA | NA | NA | NA |
|  | 5 | VSP | LR | NA | NA | NA | NA | NA | NA | NA |
|  | 6 | HWC | C | 29.4 | 31.0 | 18.8 | 10.9 | 4.9 | 0.6 | 0.0 |
|  | 6 | HWC | LR | 24.6 | 23.9 | 18.9 | 11.5 | 7.6 | 3.3 | 0.3 |
|  | 6 | VSP | C | 28.8 | 30.6 | 20.0 | 9.6 | 3.2 | 0.1 | 0.0 |
|  | 6 | VSP | LR | 22.7 | 24.5 | 17.6 | 12.5 | 6.6 | 3.2 | 0.2 |
|  | 7 | HWC | C | 31.5 | 31.5 | 20.2 | 9.2 | 2.8 | 0.0 | 0.0 |
|  | 7 | HWC | LR | 24.0 | 25.4 | 17.9 | 10.9 | 6.9 | 3.6 | 0.3 |
| 2017 | 1 | HWC | C | 21.0 | 38.1 | 18.3 | 11.8 | 0.6 | 0.0 | 0.0 |
|  | 1 | HWC | LR | 15.9 | 29.6 | 15.9 | 15.9 | 7.9 | 4.5 | 0.1 |
|  | 2 | VSP | C | 27.7 | 39.2 | 17.7 | 5.8 | 0.5 | 0.5 | 0.0 |
|  | 2 | VSP | LR | 20.8 | 28.5 | 18.2 | 10.3 | 9.3 | 2.2 | 0.5 |
|  | 3 | VSP | C | 19.4 | 43.4 | 24.8 | 10.5 | 0.0 | 0.0 | 0.0 |
|  | 3 | VSP | LR | 14.6 | 32.9 | 21.5 | 16.2 | 9.1 | 1.5 | 0.0 |
|  | 4 | VSP | C | 20.9 | 44.1 | 20.8 | 9.1 | 0.0 | 0.0 | 0.0 |
|  | 4 | VSP | LR | 14.8 | 21.1 | 21.5 | 14.7 | 8.6 | 1.3 | 0.0 |
|  | 5 | HWC | C | 30.0 | 33.1 | 16.1 | 8.4 | 0.5 | 0.0 | 0.0 |
|  | 5 | HWC | LR | 23.3 | 25.5 | 14.9 | 8.9 | 7.2 | 1.8 | 0.0 |
|  | 5 | VSP | C | 29.2 | 34.1 | 16.1 | 7.4 | 0.4 | 0.0 | 0.0 |
|  | 5 | VSP | LR | 23.6 | 26.0 | 15.5 | 9.1 | 6.0 | 1.5 | 0.0 |
|  | 6 | HWC | C | 27.2 | 36.3 | 15.4 | 7.9 | 0.6 | 0.0 | 0.0 |
|  | 6 | HWC | LR | 20.7 | 28.6 | 14.3 | 9.7 | 5.8 | 1.8 | 0.0 |
|  | 6 | VSP | C | 26.1 | 37.3 | 16.7 | 7.8 | 0.4 | 0.0 | 0.0 |
|  | 6 | VSP | LR | 19.0 | 29.6 | 16.4 | 9.2 | 6.0 | 1.4 | 0.0 |
|  | 7 | HWC | C | 27.0 | 36.0 | 15.3 | 9.1 | 1.0 | 0.0 | 0.0 |
|  | 7 | HWC | LR | 19.2 | 29.0 | 16.3 | 10.0 | 6.4 | 1.5 | 0.0 |
| ^a^HWC = High-wire cordon; VSP = Vertical shoot-positioned system.  ^b^C = Control; LR = fruiting zone leaf removal.  ^c^Veraison-to-harvest degree-hours (DH) calculated within temperature ranges of 10-15 °C, 15.1-20 °C, 20.1-25 °C, 25.1-30 °C, 30.1-35 °C, 35.1-40 °C, and >40 °C, respectively.  ^d^Data unavailable due to temperature sensor error. | | | | | | | | | | |
