# Supplementary material for: Modeling the Impacts of Weather and Cultural Factors on Rotundone Concentration in Cool-Climate Noiret Wine Grapes
Source: Front Plant Sci. 2019 Oct 15;10:1255. doi: 10.3389/fpls.2019.01255 (PMC6803480; doi:10.3389/fpls.2019.01255)
Supplement: Supplementary file 3 [file Table_3.docx]

| **Supplementary Table 3.** Fruiting zone leaf and cluster flux availability (LEFA and CEFA, respectively) of Noiret vines at the seven experimental sites measured three times during the 2016 and 2017 growing seasons. | | | | | | | | | |
| --- | --- | --- | --- | --- | --- | --- | --- | --- | --- |
| **Year** | **Site** | **Training system^a^** | **Treat-**  **ment^b^** | **LEFA*_p_*^c^ (%)** | **LEFA*_v_* (%)** | **LEFA*_r_* (%)** | **CEFA*_p_* (%)** | **CEFA*_v_* (%)** | **CEFA*_r_* (%)** |
| 2016 | 1 | HWC | C | 33.7 | 32.2 | 15.8 | 3.4 | 18.4 | 0.0 |
|  | 1 | HWC | LR | 44.1 | 53.6 | 55.0 | 38.0 | 54.0 | 54.0 |
|  | 2 | VSP | C | 34.2 | 41.7 | 27.1 | 10.2 | 24.4 | 4.8 |
|  | 2 | VSP | LR | 62.6 | 62.9 | 62.1 | 73.1 | 83.7 | 73.3 |
|  | 3 | VSP | C | 41.9 | 32.3 | 35.7 | 14.9 | 30.9 | 23.4 |
|  | 3 | VSP | LR | 58.3 | 74.7 | 57.8 | 79.3 | 80.0 | 64.4 |
|  | 4 | VSP | C | 32.6 | 37.5 | 43.2 | 12.8 | 21.8 | 23.9 |
|  | 4 | VSP | LR | 58.7 | 58.1 | 48.0 | 58.7 | 48.2 | 48.9 |
|  | 5 | HWC | C | 21.5 | 19.3 | 21.0 | 4.6 | 8.7 | 10.5 |
|  | 5 | HWC | LR | 53.3 | 49.3 | 43.1 | 52.1 | 43.0 | 44.0 |
|  | 5 | VSP | C | 33.0 | 25.7 | 28.7 | 4.4 | 3.2 | 4.2 |
|  | 5 | VSP | LR | 48.7 | 46.2 | 47.5 | 55.9 | 34.6 | 39.6 |
|  | 6 | HWC | C | 27.2 | 20.1 | 26.9 | 8.0 | 10.4 | 14.4 |
|  | 6 | HWC | LR | 46.2 | 41.9 | 44.8 | 37.6 | 37.2 | 45.4 |
|  | 6 | VSP | C | 27.4 | 27.5 | 33.6 | 23.2 | 13.6 | 21.6 |
|  | 6 | VSP | LR | 56.2 | 39.2 | 45.1 | 55.2 | 39.5 | 41.5 |
|  | 7 | HWC | C | 35.9 | 34.2 | 33.0 | 26.4 | 9.7 | 19.6 |
|  | 7 | HWC | LR | 61.0 | 62.1 | 51.4 | 51.5 | 59.9 | 42.7 |
| 2017 | 1 | HWC | C | 23.7 | 15.0 | 12.2 | 3.8 | 2.7 | 2.3 |
|  | 1 | HWC | LR | 47.5 | 67.4 | 56.5 | 50.2 | 67.4 | 57.8 |
|  | 2 | VSP | C | 38.9 | 18.6 | 23.5 | 12.1 | 1.9 | 2.8 |
|  | 2 | VSP | LR | 73.5 | 48.3 | 53.7 | 63.9 | 57.5 | 60.8 |
|  | 3 | VSP | C | 28.2 | 39.6 | 29.8 | 15.5 | 15.8 | 16.6 |
|  | 3 | VSP | LR | 51.2 | 73.9 | 65.5 | 66.1 | 58.0 | 67.8 |
|  | 4 | VSP | C | 28.6 | 38.5 | 31.2 | 23.5 | 15.9 | 17.1 |
|  | 4 | VSP | LR | 69.2 | 62.3 | 60.2 | 82.4 | 73.2 | 74.7 |
|  | 5 | HWC | C | 20.9 | 15.0 | 26.7 | 0.2 | 0.0 | 21.7 |
|  | 5 | HWC | LR | 42.8 | 31.3 | 47.8 | 46.3 | 23.3 | 45.7 |
|  | 5 | VSP | C | 29.0 | 25.6 | 30.5 | 12.9 | 11.2 | 6.4 |
|  | 5 | VSP | LR | 38.0 | 42.4 | 45.4 | 45.1 | 31.2 | 36.9 |
|  | 6 | HWC | C | 24.3 | 14.5 | 17.8 | 5.3 | 3.8 | 8.9 |
|  | 6 | HWC | LR | 40.1 | 40.2 | 44.2 | 46.0 | 38.1 | 37.4 |
|  | 6 | VSP | C | 24.0 | 18.8 | 30.2 | 13.8 | 3.4 | 8.9 |
|  | 6 | VSP | LR | 40.7 | 53.4 | 48.1 | 37.9 | 42.1 | 56.3 |
|  | 7 | HWC | C | 24.5 | 14.7 | 24.2 | 9.1 | 0.0 | 5.0 |
|  | 7 | HWC | LR | 35.8 | 38.8 | 53.1 | 39.4 | 42.4 | 57.3 |
| ^a^HWC = High-wire cordon; VSP = Vertical shoot-positioned system.  ^b^C = Control; LR = fruiting zone leaf removal.  ^c^Leaf (LEFA) and cluster (CEFA) exposure flux availability, measured at berry pea-size stage (*p*), veraison (*v*), and during grape ripening (*r*). LEFA and CEFA indicate the percentage of the above-canopy photo flux that reaches a leaf or cluster, respectively (Meyers and Vanden Heuvel, 2008). | | | | | | | | | |
