# Supplementary material for: Modeling the Impacts of Weather and Cultural Factors on Rotundone Concentration in Cool-Climate Noiret Wine Grapes
Source: Front Plant Sci. 2019 Oct 15;10:1255. doi: 10.3389/fpls.2019.01255 (PMC6803480; doi:10.3389/fpls.2019.01255)
Supplement: Supplementary file 4 [file Table_4.docx]

| **Supplementary Table 4.** Fruit composition and production metrics of Noiret vines at the seven experimental sites for the 2016 and 2017 seasons. | | | | | | | | | | | |
| --- | --- | --- | --- | --- | --- | --- | --- | --- | --- | --- | --- |
| **Year** | **Site** | **Treat-ment^a^** | **TSS**  **(°Brix)** | **pH** | **TA**  **(g/L)** | **Berry wt**  **(g)** | **Cluster wt**  **(g)** | **Cluster**  **(no./**  **vine)** | **Yield**  **(kg/m)** | **Prun. wt (kg/m)** | **Crop load**  **(kg/kg)** |
| 2016 | 1 | C | 18.4 | 3.61 | 6.36 | 1.99 | 205.9 | 14.5 | 2.98 | 0.82 | 3.64 |
|  | 1 | LR | 19.2 | 3.62 | 6.10 | 1.99 | 177.1 | 17.2 | 3.05 | 0.57 | 5.31 |
|  | 2 | C | 18.2 | 3.26 | 6.99 | 2.14 | 145.2 | 12.7 | 1.85 | 0.46 | 4.04 |
|  | 2 | LR | 20.2 | 3.33 | 6.35 | 1.95 | 109.5 | 14.1 | 1.54 | 0.67 | 2.30 |
|  | 3 | C | 20.0 | 3.46 | 6.30 | 1.97 | 181.4 | 21.3 | 3.87 | 0.54 | 7.13 |
|  | 3 | LR | 19.2 | 3.45 | 6.78 | 1.87 | 174.1 | 24.1 | 4.19 | 0.58 | 7.18 |
|  | 4 | C | 19.8 | 3.42 | 7.17 | 1.98 | 128.1 | 26.3 | 3.36 | 0.27 | 12.51 |
|  | 4 | LR | 19.4 | 3.43 | 7.37 | 1.93 | 137.4 | 26.8 | 3.68 | 0.40 | 9.18 |
|  | 5 | C | 19.8 | 3.41 | 7.92 | 1.92 | 152.3 | 44.2 | 6.72 | 0.43 | 15.56 |
|  | 5 | LR | 19.2 | 3.38 | 7.72 | 1.96 | 136.1 | 42.4 | 5.77 | 0.30 | 19.17 |
|  | 5 | C | NA^b^ | NA | NA | NA | NA | NA | NA | 0.70 | NA |
|  | 5 | LR | NA | NA | NA | NA | NA | NA | NA | 0.70 | NA |
|  | 6 | C | 21.0 | 3.5 | 7.07 | 1.94 | 133.2 | 29.3 | 3.90 | 0.67 | 5.80 |
|  | 6 | LR | 20.4 | 3.42 | 6.39 | 1.98 | 101.5 | 34.5 | 3.50 | 0.49 | 7.19 |
|  | 6 | C | 19.6 | 3.47 | 4.97 | 1.88 | 124.0 | 32.5 | 4.03 | 0.56 | 7.23 |
|  | 6 | LR | 20.2 | 3.46 | 5.25 | 2.08 | 140.7 | 28.7 | 4.04 | 0.45 | 8.95 |
|  | 7 | C | 20.2 | 3.56 | 5.25 | 1.78 | 99.5 | 16.1 | 1.61 | 0.32 | 5.02 |
|  | 7 | LR | 20.8 | 3.52 | 5.69 | 1.61 | 104.1 | 25.2 | 2.62 | 0.27 | 9.83 |
| 2017 | 1 | C | 18.0 | 3.4 | 9.57 | 1.74 | 159.1 | 29.0 | 4.61 | 0.95 | 4.83 |
|  | 1 | LR | 18.7 | 3.37 | 6.86 | 1.64 | 127.3 | 29.0 | 3.69 | 0.45 | 8.23 |
|  | 2 | C | 16.9 | 3.24 | 7.75 | 1.96 | 112.2 | 16.8 | 1.88 | NA^c^ | NA |
|  | 2 | LR | 17.8 | 3.19 | 7.49 | 1.94 | 115.1 | 22.4 | 2.58 | NA | NA |
|  | 3 | C | 19.2 | 3.46 | 7.21 | 1.68 | 146.9 | 38.3 | 5.62 | 0.81 | 6.93 |
|  | 3 | LR | 18.2 | 3.37 | 7.36 | 1.79 | 124.6 | 35.2 | 4.38 | 0.66 | 6.59 |
|  | 4 | C | 18.1 | 3.53 | 6.99 | 2.44 | 131.7 | 39.3 | 5.18 | 0.42 | 12.37 |
|  | 4 | LR | 18.0 | 3.62 | 7.62 | 2.20 | 136.4 | 30.1 | 4.10 | 0.45 | 9.18 |
|  | 5 | C | 17.1 | 3.45 | 8.77 | 1.79 | 133.2 | 30.6 | 4.08 | 0.39 | 10.43 |
|  | 5 | LR | 17.8 | 3.3 | 8.89 | 1.77 | 99.1 | 53.0 | 5.25 | 0.29 | 18.30 |
|  | 5 | C | 18.2 | 3.23 | 8.56 | 1.89 | 151.2 | 37.3 | 5.64 | 0.36 | 15.88 |
|  | 5 | LR | 18.0 | 3.39 | 8.99 | 1.80 | 125.4 | 51.8 | 6.49 | 0.26 | 25.28 |
|  | 6 | C | 19.1 | 3.39 | 8.31 | 2.01 | 144.5 | 26.2 | 3.79 | 0.93 | 4.08 |
|  | 6 | LR | 19.8 | 3.23 | 8.23 | 1.95 | 115.4 | 34.2 | 3.94 | 0.60 | 6.53 |
|  | 6 | C | 19.0 | 3.33 | 8.61 | 1.85 | 112.1 | 34.0 | 3.81 | 0.92 | 4.17 |
|  | 6 | LR | 20.4 | 3.41 | 7.84 | 1.76 | 112.3 | 39.9 | 4.48 | 0.61 | 7.35 |
|  | 7 | C | 19.6 | 3.38 | 9.02 | 2.00 | 121.4 | 32.5 | 3.95 | 1.04 | 3.78 |
|  | 7 | LR | 19.2 | 3.62 | 7.98 | 1.89 | 142.9 | 27.3 | 3.91 | 0.99 | 3.95 |
| ^a^C = Control; LR = fruiting zone leaf removal.  ^b^Data unavailable due to commercial harvest of experimental fruit.  ^c^Data unavailable due to commercial dormant pruning of experimental vines. | | | | | | | | | | | |
