# Supplementary material for: Modeling the Impacts of Weather and Cultural Factors on Rotundone Concentration in Cool-Climate Noiret Wine Grapes
Source: Front Plant Sci. 2019 Oct 15;10:1255. doi: 10.3389/fpls.2019.01255 (PMC6803480; doi:10.3389/fpls.2019.01255)
Supplement: Supplementary file 5 [file Table_5.docx]

| **Supplementary Table 5.** Leaf petiole macronutrient concentrations and berry water status (via δ^13^C) of Noiret vines at the seven experimental sites for the 2016 and 2017 seasons. | | | | | | | | | |
| --- | --- | --- | --- | --- | --- | --- | --- | --- | --- |
| **Year** | **Site** | **Treatment^a^** | **N**  **(%)** | **P**  **(%)** | **K**  **(%)** | **Mg**  **(%)** | **Ca**  **(%)** | **δ^13^C**  **(‰)** |  |
| 2016 | 1 | C | 1.00 | 0.19 | 2.61 | 0.26 | 1.06 | -28.6 |  |
|  | 1 | LR | 1.07 | 0.24 | 3.14 | 0.21 | 1.08 | -29.1 |  |
|  | 2 | C | 0.88 | 0.54 | 1.10 | 0.84 | 1.25 | -29.4 |  |
|  | 2 | LR | 0.89 | 0.52 | 1.25 | 0.68 | 1.30 | -28.3 |  |
|  | 3 | C | 0.75 | 0.13 | 0.93 | 1.32 | 1.82 | -28.0 |  |
|  | 3 | LR | 0.85 | 0.15 | 1.07 | 1.03 | 1.82 | -28.4 |  |
|  | 4 | C | 0.92 | 0.23 | 1.18 | 0.83 | 2.47 | -26.2 |  |
|  | 4 | LR | 1.03 | 0.26 | 2.29 | 0.6 | 2.40 | -26.3 |  |
|  | 5 | C | 1.06 | 0.29 | 2.74 | 0.60 | 1.63 | -27.2 |  |
|  | 5 | LR | 0.97 | 0.24 | 1.78 | 0.82 | 1.76 | -28.1 |  |
|  | 5 | C | 1.04 | 0.18 | 2.81 | 0.59 | 1.78 | NA^b^ |  |
|  | 5 | LR | 1.08 | 0.23 | 3.23 | 0.58 | 1.91 | NA |  |
|  | 6 | C | 0.76 | 0.27 | 1.41 | 0.83 | 2.06 | -26.2 |  |
|  | 6 | LR | 0.64 | 0.32 | 1.23 | 0.93 | 2.41 | -27.2 |  |
|  | 6 | C | 0.69 | 0.53 | 0.70 | 1.35 | 2.45 | -26.2 |  |
|  | 6 | LR | 0.70 | 0.37 | 0.82 | 1.18 | 2.28 | -27.6 |  |
|  | 7 | C | 0.86 | 0.13 | 1.92 | 0.68 | 2.29 | -25.2 |  |
|  | 7 | LR | 0.83 | 0.10 | 1.74 | 0.69 | 2.07 | -24.8 |  |
| 2017 | 1 | C | 1.17 | 0.34 | 2.53 | 0.27 | 1.19 | -28.5 |  |
|  | 1 | LR | 0.95 | 0.35 | 3.23 | 0.31 | 1.68 | -28.8 |  |
|  | 2 | C | 0.75 | 0.59 | 0.79 | 0.78 | 1.33 | -29.4 |  |
|  | 2 | LR | 0.72 | 0.62 | 0.82 | 0.91 | 1.72 | -29.1 |  |
|  | 3 | C | 1.12 | 0.17 | 0.86 | 0.99 | 1.88 | -27.3 |  |
|  | 3 | LR | 1.17 | 0.17 | 1.53 | 0.72 | 2.08 | -27.9 |  |
|  | 4 | C | 0.78 | 0.38 | 2.04 | 0.39 | 1.99 | -27.7 |  |
|  | 4 | LR | 0.76 | 0.46 | 2.48 | 0.30 | 1.99 | -27.9 |  |
|  | 5 | C | 1.01 | 0.24 | 2.34 | 0.85 | 1.95 | -27.1 |  |
|  | 5 | LR | 1.01 | 0.32 | 1.79 | 1.01 | 1.96 | -28.2 |  |
|  | 5 | C | 0.91 | 0.19 | 2.11 | 0.54 | 1.83 | -27.9 |  |
|  | 5 | LR | 0.92 | 0.26 | 2.62 | 0.58 | 2.01 | -28.5 |  |
|  | 6 | C | 0.81 | 0.33 | 0.93 | 0.63 | 2.02 | -28.7 |  |
|  | 6 | LR | 0.68 | 0.48 | 0.63 | 1.05 | 2.83 | -29.2 |  |
|  | 6 | C | 0.67 | 0.52 | 0.83 | 0.94 | 2.14 | -29.7 |  |
|  | 6 | LR | 0.72 | 0.49 | 0.53 | 1.15 | 2.51 | -29.1 |  |
|  | 7 | C | 0.82 | 0.27 | 1.33 | 0.66 | 1.93 | -28.4 |  |
|  | 7 | LR | 0.74 | 0.19 | 1.01 | 0.72 | 2.04 | -29.6 |  |
| ^a^C = Control; LR = fruiting zone leaf removal.  ^b^Data unavailable due to commercial harvest occurring prior to harvest of experimental vines. | | | | | | | | | |
